# The role of sweet/fruit-flavored disposable electronic cigarettes on early nicotine initiation - a systematic review

**DOI:** 10.1186/s12889-025-21897-z

**Published:** 2025-02-17

**Authors:** Paulina Natalia Kopa-Stojak, Rafal Pawliczak

**Affiliations:** Department of Immunopathology, Faculty of Medicine, Medical University of Lodz, Lodz, Poland

**Keywords:** Disposable e-cigarette, Cig-a-like e-cigarette, Early nicotine initiation, Sweet/ fruit-flavored e-liquid, Youths

## Abstract

**Background:**

Sweet/fruit disposable e-cigarettes (ECs) are cheap, easy to use, and look like inconspicuous colored markers, which encourages young people and young adults to use them. This work attempts to summarize current knowledge about the effect of sweet/fruit-flavor disposable ECs on early nicotine initiation.

**Methods:**

The literature search was performed in June 2024 in Pub Med, Scopus, Web of Science and Science Direct databases by the terms ‘ends’, ‘electronic nicotine delivery system’, ‘disposable electronic cigarette’, ‘disposable e-cigarette’, ‘cig-a-like e-cigarette’, ‘cig-a-like electronic cigarette’, ‘nicotine initiation’.

**Results:**

This systematic review analyzes findings from four heterogenous US studies. All analyzed studies highlighted that sweet/fruit-flavored ECs, compared to mint/menthol- or tobacco-flavors, were the most commonly chosen by youths and young adults during initiation and progression of vaping, regardless of the device type. Furthermore, two studies determined that never-smokers mostly start vaping by using modifiable ECs compared to disposable EC devices. Moreover, all studies showed that initiation by using disposable ECs was lower in people who had never previously vaped or smoked compared to current/former ECs users and former tobacco cigarettes (TCs) smokers or dual users.

**Conclusions:**

Due to limited number of studies, their limited location, scope (mostly ever users aged ≥ 18), and moderate quality of the studies, it is difficult to clearly determine the effect of sweet/fruit-flavored disposable ECs on early nicotine initiation. Moreover, it is difficult to determine if sweet/fruit-flavored disposable EC have a role in the avoiding the initiation of tobacco cigarettes or delaying the initiation by the effect of competition. The findings from this systematic review are preliminary and require validation through high-quality, global studies among youth and young adult never-smokers/never-vapers who initiated using tobacco products with sweet/fruit-flavored disposable ECs and continue vaping and/or smoking any tobacco products (and any flavors).

**Trial registration:**

The study protocol of this systematic review was registered in International Prospective Register of Systematic Reviews (PROSPERO) with registration number CRD42024585153.

**Supplementary Information:**

The online version contains supplementary material available at 10.1186/s12889-025-21897-z.

## Introduction

Electronic nicotine delivery systems (ENDS) are the battery-operated devices which heat e-liquid to create aerosol inhaled by the users [1]. Global market of electronic cigarettes (e-cigarettes or ECs) reached USD 28.18 billion in 2023 and is expected to grow by 30.6% according to compound annual growth rate (CAGR) from 2023 to 2030 [2]. E-cigarettes are rising in popularity both among youths and adults [3, 4]. According to statistics, there were 58.1 million of vapers in 2018 worldwide. This number increased to 68 million of ECs users in 2020 [5] and was estimated to raise to 86.1 million in 2023 [6].

There are some significant differences in preferences of flavors, nicotine content and reasons of choosing ECs by youths and adults. Adolescents (< 18 years old) and young adults (18–24 years old) have more intention to initiate vaping ECs with sweet, fruit, coffee, menthol-flavored liquids compared to tobacco-flavored liquids [7–10]. In addition, adolescent never smokers are more likely to try sweet- and/or fruit-flavored ECs than smokers who want to quit smoking [11, 12]. Moreover, adolescent and young adult never smokers mostly preferred nicotine-free or low-nicotine ECs, and young smokers have willingness to use medium- and high-nicotine ECs [13, 14]. Young people mostly choose ECs because of modern design, access to thousands of flavors (about 7500 [15]) and different nicotine concentrations. In addition, they perceive ECs as harmless or less harmful when compared to conventional tobacco cigarettes (TCs) [16–18]. Adult smokers (> 24 years old) were more interested in tobacco-flavored ECs [13, 19] with nicotine concentration of 18 mg/mL [20]. Furthermore, adult smokers use ECs mostly as an aid to stop smoking, a safer alternative than combustible cigarettes or a way to circumvent tobacco-free area regulations [21, 22].

Scientists are still learning about short- and long-term health effects of smoking ECs [23]. Moreover, aerosol from sweet/fruit-flavored ECs may contain lower doses (compared with conventional cigarettes) of: carbonyls (i.e. formaldehyde, acetaldehyde, acetone, propionaldehyde, acrolein, diacetyl), polycyclic aromatic hydrocarbons (PAHs) (i.e. naphthalene, chrysene, benzo(a)anthracene, 2,3-pentadiene), aromatic amines (i.e. 2-aminonaphtalene, 3- and 4- aminobiphenyls, o-toluidine), metals (i.e. chromium, iron and zinc) [24, 25]. Furthermore, exposure to ENDS aerosol and e-liquids leads to oxidative stress and inflammatory responses, impaired endothelial function, increased platelet activation and aggregation, elevated vascular stiffness and blood pressure, and altered gene expression [26–28].

Since 2020 there has been an exponential increase in the sales of disposable e-cigarettes (especially sweet-flavored) among young people. According to the 2020 USA National Youth Tobacco Survey, there was a significant increase in disposable e-cigarettes use among middle and high school students (from 5.4% in 2019 (corresponding to 120,000 students) to 41.7% (corresponding to 870,000 students) in 2020) [29]. Low prices, wide range of flavors (tobacco, menthol/mint, fruit, desserts, drinks), design (look like colorful markers) and persuasive promotion in social networks have made them increasingly popular also among non-smokers who chose disposable ECs as initial smoking devices [30, 31]. Aerosol from disposable flavored e-cigarettes may contain some hazardous and potentially hazardous constituents (HPHCs), including flavoring agents, carbonyls, volatile organic compounds, metals and reactive oxygen species (ROS) [32–38]. In addition, aerosol of some disposable ECs may contain higher nicotine content (3.1–6.7 mg/15 puffs vs. 1.7 mg/15 puffs) than JUUL device (max. nicotine concentration in JUUL device: 20 mg/mL in EU and 56 mg/mL in US) [35]. The following systematic review attempts to summarize current knowledge about the effects of sweet/fruit-flavored disposable e-cigarettes on early nicotine initiation.

## Methods

### Eligibility criteria

The study protocol of this systematic review was registered in International Prospective Register of Systematic Reviews (PROSPERO) with registration number CRD42024585153, and was carried out in accordance with Preferred Reporting Items for Systematic Reviews and Meta-Analyses (PRISMA) guidelines [39]. This systematic review analyzes the effect of sweet/fruit-flavored disposable e-cigarettes on nicotine initiation. The publications selection was based on pre-determined inclusion and exclusion criteria. The inclusion criteria of this systematic review were: study type (controlled, cohort, cross-sectional, observational, prospective), age of participants (adolescents (12–17 years-old) and young adults (18–24 years-old)), smoking status (never-smokers), EC device type (disposable), e-liquid flavor (sweet/fruit), measuring outcomes (initiation of use, continuation of use) and study language (English).

### Search strategy

The literature search in PubMed, Scopus, Web of Science and Science Direct databases was performed up to July 25, 2024 (without time limits of publications). In addition, the reference list of included human studies was reviewed to add other relevant studies. The combination of the search terms was: [electronic AND nicotine AND delivery AND system OR ends OR disposable AND electronic AND cigarette OR disposable AND e-cigarette OR cig-a-like AND electronic AND cigarette OR cig-a-like AND e-cigarette] AND [nicotine AND initiation] for PubMed and Scopus databases. In the case of Web of Science database, because of the great number of searching results (about 2.9 million of results), the search terms were narrowed to: [ends OR disposable electronic cigarette OR disposable e-cigarette OR cig-a-like electronic cigarette OR cig-a-like e-cigarette] AND [nicotine initiation]. Similarly, in Science Direct the search terms were narrowed to: [disposable electronic cigarette OR disposable e-cigarette OR cig-a-like electronic cigarette OR cig-a-like e-cigarette] AND [nicotine initiation]. Full search strategy for each database is presented in *Additional file 1*.

### Study selection

This systematic review focuses on human studies that analyzed the effect of sweet/fruit-flavored disposable e-cigarettes on nicotine initiation among adolescents and young adults. After removing duplicate articles between databases, both authors (P.N. K-S. and R. P.) conducted an independent selection of publications by the title and abstract to select potentially relevant human studies to be further analyzed in this systematic review. The exclusion criteria of irrelevant articles were: publication type (letter to the editor, commentary, review/systematic review, protocols), language (other than English), EC device type (other than disposable, i.e. pod, tank, cartridge ECs), e-liquids (other than sweet/fruit flavors, i.e. tobacco, mint/menthol), age of EC users (adults) and scope of publication (chemical composition, analytical methods, tobacco market size estimations, regulatory frameworks).

Then, full versions of articles initially selected from databases by the title and abstract were thoroughly analyzed to choose the most relevant ones to be further included in the review process. At this stage, the studies which analyzed ECs in general, without division into device type (disposable/prefilled), into e-liquid flavor (sweet/fruit, mint/menthol, tobacco, unflavored, other), into participants age groups (adolescents, young adults, adults) or other unrelated studies were excluded from further review. Consensus in the case of any discrepancies between the authors’ opinions on relevance of some individual studies and their inclusion into further investigation (based on the inclusion and exclusion criteria) was achieved by discussion.

### Data extraction and synthesis

The key data extracted from each relevant study (based on inclusion/exclusion criteria) were: author(s) and the year of publication, study design, study location, sample size, age and sex of the participants, measured variables, main findings and study limitations. Then, the data were synthesized for further analysis and presented in narrative form.

### Quality assessment of included studies

The quality assessment of included studies for this systematic review was carried out with NIH Study Quality Assessment Tools [40]. The tools were designed specifically to certain study designs (controlled intervention, observational cohort, cross-sectional, case control, pre-post, case series studies) and tested for any potential flaws in study methods and/or implementations. The Study Quality Assessment Tools consist of 14 questions with possible answers: “yes”, “no”, “not reported”, “cannot be determined” or “not applicable”. “Cannot be determined” and “not applicable” were noted as representing potential flaws. The quality of each study may be reported as poor (0–4 out of 14), fair (5–10 out of 14) or good (11–14 out of 14) [41].

## Results

### Study selection

**Fig. 1:**
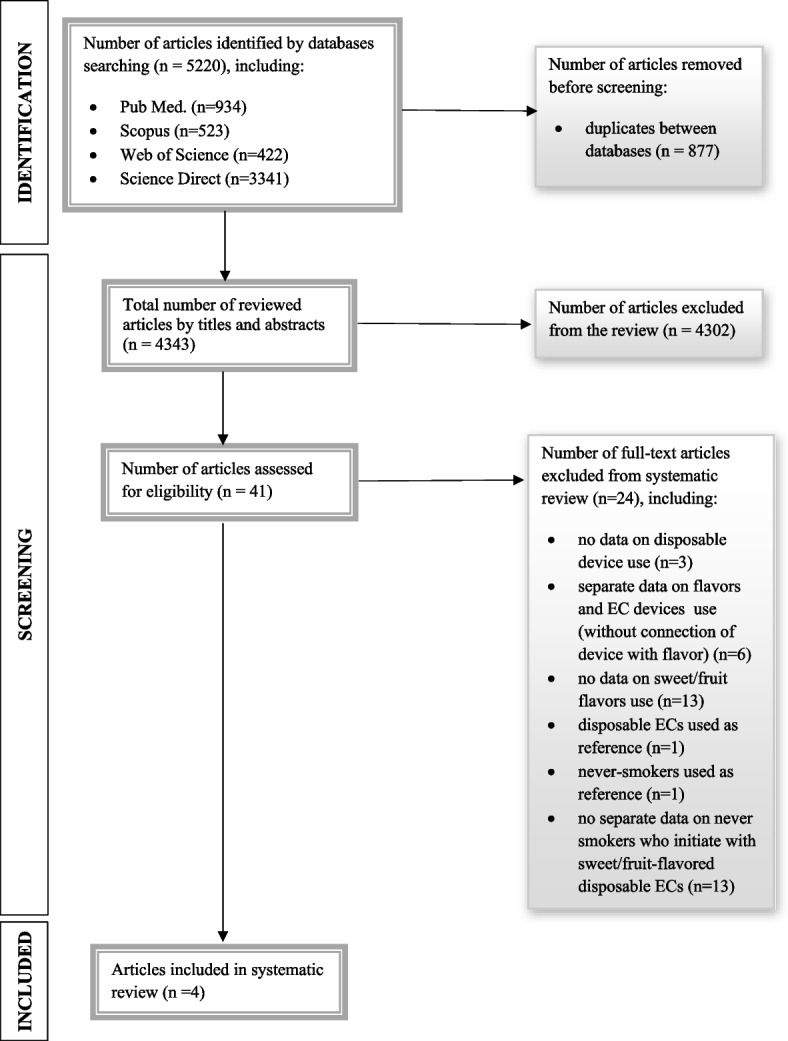
Article selection process recommended by the Preferred Reporting Items for Systematic Reviews and Meta-Analyses (PRISMA) statement

The literature search allowed to identify *n* = 5220 potential articles (including *n* = 934 in Pub Med, *n* = 523 in Scopus, *n* = 422 in Web of Science and *n* = 3341 in Science Direct database). Removing duplicate publications between the searched databases allowed to reduce the number of publications to *n* = 4343, which were analyzed by the title and abstract. Then, the number of articles was reduced to 41, which were assessed for eligibility. Finally, *n* = 4 studies were included in this systematic review. Searching strategy of article identification, as recommended by Preferred Reporting Items for Systematic Reviews and Meta-Analyses (PRISMA) Statement is presented in Fig. 1.

### Study characteristics

Study characteristics, main findings and limitations are presented in Table 1. All publications included in this systematic review [12, 42–44] were based on research samples from the United States. This makes it difficult to generalize the obtained data to the whole world population, due to unique US marketing and regulatory dynamics. Furthermore, the vast majority of the publications were published between 2021 and 2023. Due to the epidemic, the level of ECs use by youths and popularity of certain products (including fruit and mint flavors), in 2020 Food and Drug Administration (FDA) implemented enforcement policy on unauthorized flavored cartridge-based e-cigarettes, which significantly changed the dynamics of ECs market. Three of the publications included in this systematic review were published after the implementation of the enforcement policy, and only one [12] before this regulation had been implemented.

**Table 1:** Characteristics, main findings and limitations of four US studies included in the systematic review

Author(s), year of publication	Study design	Participants	Sample size	Measured variables	Main findings	Limitations
(Leventhal et al*.* 2021) [42]	Prospective cohort data from The Happiness & Health study (2018–2019)	9th grade high school students	n = 3396 (baseline) and n = 266 (follow up)	• Demographic information • Disposable ECs use (P30D) • Most used flavor • Disposable and rechargeable EC dual using (P30D) • Vaping: dependence symptoms, onset in high school, to quit smoking • TCs use (never/former/current) • Rechargeable ECs use (never/former/current)	• Initiation of disposable ECs use was higher among baseline former (22.1%) and current (50.2%) versus never (6.3%) rechargeable ECs users • Initiation of disposable ECs use was higher in baseline never-vaping former smokers compared with never-users of any tobacco product (18.2% vs. 5.7%; aOR = 3.9; 95% CI: 2.1–7.5) • Initiation of disposable ECs use was higher in baseline current dual users compared with never-smoking current vapers (61.3% vs. 42.2%; aOR = 3.0; 95% CI: 1.5–6.0) (stratified analysis) • Among follow-up current disposable ECs users ice-flavored (vs. fruit/sweet-flavored) ECs use (adjusted rate ratio (aRR) = 1.5; 95% CI:1.0–2.1) was cross-sectionally associated with more past-month disposable ECs use days • Among follow-up current disposable ECs users vaping dependence symptoms (aRR = 2.2; 95% CI: 1.5–3.2) were cross-sectionally associated with more past-month disposable ECs use days	• Results based on the self-reported tobacco product use behaviors (risk of response bias) • Study sample from Los Angeles, CA (unknown generalizability) • Relatively small cell size for some stratified analyses (no statistical significance) • Follow up carried out during COVID-19 pandemic (may have impacted youth tobacco/ECs use)
(Crespi et al*.* 2023) [43]	A longitudinal cohort VAPER study (Waves 2 and 3; December 2020-December 2021)	Young adults and adults (≥ 21 years-old)	n = 1809	• Demographic information • Smoking status (current, former, never) • EC devices use • Liquid type use (nicotine salt, freebase) • Reasons for continued ECs use • Flavors used • Additional measures (for device types and flavors): nicotine concentration, wattage, voltage, resistance, age of EC first use	• Among the users of disposable devices with nicotine salt liquid, 90.2% used sweet, 9.7% mint/menthol, 0.1% tobacco flavor • 90.4% of ECs users continued vaping because of the joy from the flavor used sweet flavors, compared with ECs users, who continued for other reasons (73.5%; *p* < 0.001) • In disposable devices with nicotine salt liquid nicotine concentration was 55.5 mg/mL (tobacco-flavored), 50.7 mg/mL (mint/menthol-flavored), 50.5 mg/mL (sweet-flavored) • Disposable device with nicotine salt liquid ECs power was: 11.1W (mint/menthol-flavored), 12.2W (sweet-flavored) • Disposable device with nicotine salt liquid ECs voltage was: 4 V (mint/menthol-flavored), 4.1 V (sweet-flavored) • Disposable device with nicotine salt liquid ECs resistance was: 1.6 Ohms (mint/menthol-flavored), 1.5 Ohms (sweet-flavors) • Disposable device with nicotine salt liquid EC first use was: 60 years (tobacco-flavored), 28.1 years (mint/menthol-flavored), 26.8 years (sweet-flavor)	• Results based on the self-reported tobacco products use behaviors (risk of response bias) • USA study (difficulties with results generalization) • Some parts of the study was carried out during COVID-19 pandemic (may have impacted youth tobacco/ECs use) • No data for users 12–17 years old (adolescents) and 18–20 years old (young adults)(only data for young adults aged 21–24 years old and adults aged ≥ 25 years old)
(Farsalinos et al*.* 2023) [44]	An online survey	Young adults and adults (≥ 18 years old)	*n* = 69233	• Demographic information • Smoking status (never/former/current) • First EC device use • Nicotine concentration use • Flavor choice, accessibility and frequency of using • ECs use for quitting smoking (patterns, devices, flavors, nicotine concentration)	• 13.4% of participants currently smoke TCs, 81.3% are former smokers, 5.2% are never-smokers • 8.1% of participants initiated ECs use with disposable devices • Among never-smokers, who initiated ECs use, 81.9% chose fruit, 69.4% desserts, 56.9% candy/sweet, 24.2% coffee, 20.8% non-alcoholic/non-coffee cocktails, 18.7% menthol, 14.9% mint, 10.4% tobacco, 9.2% alcoholic cocktails, 1.8% unflavored and 20% other flavors • 0.2% of participants continued ECs use with disposable devices • Only 1% of never smokers continued EC use with disposable devices • Among never smokers, who continued ECs use, 81.2% chose fruit, 60.2% desserts, 43.1% candy/sweet, 16.3% coffee, 15.4% menthol, 13.1% non-alcoholic/non-coffee cocktails, 11.4% mint, 8.5% spice, 6.8% alcoholic cocktails, 5.2% tobacco, 1.2% unflavored and 10.8% other flavors • At a time of quitting smoking, 85.1% of participants used ECs every day, 6.6% some days and 8.3% not at all • At a time of quitting smoking, 1.9% of participants chose disposable devices	• Results based on the self-reported tobacco products use behaviors (risk of response bias) • USA study (difficulties in generalization) • Only separate data for never-smokers (TC and EC never-smokers) who began with fruit/sweet-flavors • Only separate data for never-smokers (TC and EC never-smokers) who continued disposable ECs use with fruit/sweet-flavors (separate data for disposable device continued use and separate data for continued use of sweet/fruit flavors among never-smokers) • No data for users 12–17 years old (data for young adults aged 18–24 years old and adults aged ≥ 25 years old)
(Shang et al*.* 2018) [12]	An online discrete choice experiment (2015)	Adolescents (14–17 years-old)	n = 515	• Demographic information • Smoking status • EC devices use • Flavors use • FDA warning on ECs	ECs never-users who: • Chose sweet/fruit flavors showed significantly (*p* < 0.01) increased likelihood of ECs use • Chose mint/menthol flavors showed significantly (*p* < 0.05) increased likelihood of further ECs use • Chose modifiable devices (vs. disposable devices) showed significantly (*p* < 0.05) increased likelihood of further ECs use	• Results based on the self-reported tobacco products use behaviors (risk of response bias) • USA study (difficulties in generalization) • Cig-a-likes (disposable ECs) used as a reference group in the analysis of the probability of EC use by never-users

*Abbreviations: aOR* adjusted odds ratio, *aRR* adjusted risk ratio, *CI* confidence interval, *EC* electronic cigarette, *FDA* Food and Drug Administration, *P30D* past 30 days, *TC* tobacco cigarette

### Quality of the studies

Quality of the studies included in this systematic review is presented in Table 2. The NIH Quality Assessment Tool used for each of the study assessed the score range between six to eight (one study scored at 8, two at 7 and one at 6), which is classified as fair quality of the studies. All of the studies based on the participants self-reported data on retrospective studies, which may reduce the reliability and measurement validity for dependent and independent variables, and increase the probability of bias. In addition, the participation rate of eligible persons cannot be determined. Only two out of four studies clearly defined inclusion and exclusion criteria, and only one justified the sample size. Moreover, two of the studies presented results for baseline and follow-up. None of the studies has outcome assessors blinded to the exposure of the participants and two of them measure the impact of potential confounding variables on the relationship between exposure and outcome.

**Table 2:** Quality assessment of the studies by NIH study quality assessment tool

		Studies (author(s), year)			
#	Criteria	Leventhal et al., 2021 [42]	Crespi et al., 2023 [43]	Farsalinos et al., 2023 [44]	Shang et al., 2018 [12]
1	**Was the research question or objective in this paper clearly stated?**	Yes	Yes	Yes	Yes
2	**Was the study population clearly specified and defined?**	Yes	Yes	Yes	Yes
3	**Was the participation rate of eligible persons at least 50%?**	CD	CD	CD	CD
4	**Were all the subjects selected or recruited from the same or similar populations (including the same time period)? Were inclusion and exclusion criteria for being in the study prespecified and applied uniformly to all participants?**	CD	Yes	Yes	CD
5	**Was a sample size justification, power description, or variance and effect estimates provided?**	NR	NR	NR	Yes
6	**For the analyses in this paper, were the exposure(s) of interest measured prior to the outcome(s) being measured?**	Yes	Yes	Yes	Yes
7	**Was the timeframe sufficient so that one could reasonably expect to see an association between exposure and outcome if it existed?**	Yes	Yes	Yes	Yes
8	**For exposures that can vary in amount or level, did the study examine different levels of the exposure as related to the outcome (e.g., categories of exposure, or exposure measured as continuous variable)?**	Yes	Yes	Yes	Yes
9	**Were the exposure measures (independent variables) clearly defined, valid, reliable, and implemented consistently across all study participants?**	No	No	No	No
10	**Was the exposure(s) assessed more than once over time?**	Yes	NR	Yes	NR
11	**Were the outcome measures (dependent variables) clearly defined, valid, reliable, and implemented consistently across all study participants?**	No	No	No	No
12	**Were the outcome assessors blinded to the exposure status of participants?**	NR	NR	NR	NR
13	**Was loss to follow-up after baseline 20% or less?**	Yes	NA	NA	NA
14	**Were key potential confounding variables measured and adjusted statistically for their impact on the relationship between exposure(s) and outcome(s)?**	Yes	Yes	No	No
**Total score**		**8**	**7**	**7**	**6**

*Abbreviations: CD* cannot determine, *NA* not applicable, *NR* not reported

### Association between the use of sweet/fruit-flavored disposable e-cigarettes and nicotine initiation among youths and young adults

The prospective cohort data from the Happiness & Health study carried out among high school students (9th grade) showed that initiation of disposable ECs was higher among baseline former (in 56/253 (22.1%) cases) and current (in 130/259 (50.2%) cases) than among never (in 87/1391 (6.3%) cases) rechargeable ECs users. In addition, stratified analysis showed that initiation of disposable ECs was higher in people who had never previously used e-cigarettes (in 76/234 (32.5%) cases) compared to never users of any tobacco product (18.2% vs. 5.7%; aOR = 3.9; 95% CI: 2.1–7.5) and in current dual users than in never-smokers currently using e-cigarettes (61.3% vs. 42.2%; aOR = 3.0; 95% CI: 1.5–6.0). In addition, ice (in 144/266 (54.3%) cases), followed by sweet/fruit (in 58/266 (21.9%) cases) and mint/menthol (in 39/266 (14.7%) cases) flavors were the most commonly chosen by current disposable ECs users. During follow-up of the study, among current disposable ECs users, ice-flavored (vs. fruit/sweet-flavored) ECs use (adjusted rate ratio (aRR) = 1.5; 95% CI:1.0–2.1) and e-cigarette use dependence symptoms (aRR = 2.2; 95% CI: 1.5–3.2) was cross-sectionally associated with more past-month disposable ECs use days. Unfortunately, that study focused only on disposable ECs use (initiation and follow-up) and there is no data on the number of participants who started with sweet/fruit-flavored disposable ECs and progressed smoking with other tobacco products (i.e. rechargeable ECs or combustible cigarettes) [42].

In a longitudinal cohort VAPER study of U.S. ≥ 21 years old ENDS users, 79.8% of never-smokers (in 214/263 cases) chose sweet flavors, compared with 14.2% of menthol/mint (in 36/263 cases) and 6.1% of tobacco flavors (in 13/263 cases). Among disposable devices with nicotine salt liquid users, 90.2% used sweet (in 308/341 cases), 9.7% mint/menthol (in 32/341 cases) and 0.1% tobacco flavor (in 1/341 cases). Furthermore, among sweet/fruit-flavored liquids, nicotine salt concentrations were the highest in disposable devices (50.5 mg/mL), compared to disposable pod/cartridge devices (38.2 mg/mL) and refillable pod/cartridge devices (37.7 mg/mL). There was similar age of the first trying of sweet/fruit-flavored disposable ECs compared with refillable pod/cartridge ECs (26.8 vs. 26.7 years) but higher than for disposable pod/cartridge ECs (24.7 years). In addition, 90.4% of ECs users (172/192 cases) who continued using e-cigarettes because of the joy of the taste used sweet flavors, compared with ECs users who continued vaping for other reasons (73.5% (963/1297 cases); *p* < 0.001). However, the study population focused on ECs users aged ≥ 21 years old, and we have no data for adolescents (12–17 years-old) but only for young adults aged 21–24 (no data for users 18–20 years old) and adults aged ≥ 25 years old. In addition, the data are divided by age into two groups: 21–29 years old (young adults and adults) and ≥ 30 years old (adults). Therefore, basing on partial data only for 21–24 years old users (mixed with adults 25–29 years old), it is hard to appropriately analyze the effect of sweet/fruit-flavored disposable ECs on early nicotine initiation [43].

An online cross-sectional study of ≥ 18 years old ECs ever users (including 5.2% of never-smokers (3,600/69,233), 13.4% current smokers (9,277/69,233) and 81.3% former smokers (56,286/69,233)) showed that 8.1% of participants (5,635/69,233) initiated smoking by using disposable ECs. Never-smokers initiated ECs mostly with fruit (in 2,948/3,600 (81.9%) cases), dessert (in 2,498/3,600 (69.4%) cases) or candy/sweet (in 2,048/3,600 (56.9%) cases) flavors by choosing mostly personalized vaporizers (in 2,462/3,600 (68.4%) cases) instead of disposable (in 36/3600 (1%) cases) ECs device. In addition, fruit (in 4,666/5,635 (82.8%) cases), followed by dessert (in 3,866/5,635 (68.6%) cases) and sweet (in 2,941/5,635 (52.2%) cases) were the most common initial flavors used regularly by disposable ECs users. Furthermore, disposable ECs continued to be used by 0.2% of study participants (in 155/69,233 cases), usually with fruit (in 129/155 (83%) cases), dessert (in 109/155 (70.5%) cases) or sweet (in 72/155 (46.3%) cases) e-liquid flavors. However, there is no data on the number of never-smoker participants who started with sweet/fruit-flavored ECs and continued with using any other tobacco products. Furthermore, the study population focused on ECs users ≥ 18 years old, including young adults and adults. Moreover, there is no data for adolescents (12–17 years old) and no division into age groups (no separate data for young adults and adults; mean age of study participants: 34.6 ± 11.6 years-old) [44].

Finally, an online discrete choice experiment conducted in 2015 showed higher probability of choosing ECs with fruit/sweet flavors (*p* < 0.1 for ever-users; *p* < 0.01 for never-users) compared to tobacco flavor. In addition, there was higher probability of using ECs among never-users (*p* < 0.05) for modifiable devices (i.e. eGo/Mods/APVs) compared to cig-a-likes. However, this study based on the data collected in 2015, where there was no access to modern disposable ECs devices (only cig-a-like, 1st generation ECs) and the popularity of cig-a-like ECs was low compared to modifiable devices. Therefore, cig-a-likes were used as a reference group in the analysis of the probability of using ECs by ever- and never-users [12].

## Discussion

Our systematic review showed that the sweet/fruit-flavored ECs compared to mint/menthol-flavored or tobacco-flavored ECs were the most commonly chosen by youths and young adults during initiation and progression of smoking, regardless of the device type. In addition, most ECs users (including disposable devices users) continued using them because of the joy of sweet/fruit taste. Furthermore, never-smokers mostly started smoking ECs with advanced personalized vaporizers, disposable devices were less common. Moreover, initiation of using disposable ECs was lower in never-smokers group compared to current/former ECs smokers and former tobacco cigarettes (TCs) smokers or dual users. However, basing on the studies included in this systematic review, we are unable to clearly determine the effect of sweet/fruit-flavored disposable ECs on early nicotine initiation due to limited scope of the studies, their number, study population (US only) and quality.

Disposable electronic cigarettes were described by the youth users as a ‘cool,’ ‘fashionable,’ ‘modern lifestyle accessory,’ ‘brightly colored devices with range of flavorings,’ ‘designed in a way to target youths’ [45]. Nielsen retail scanner data analysis determined a nearly ten-fold increase in disposable products sales from July 2019 to July 2020, dominated by fruit-flavored ECs [46]. A cross-sectional survey of U.S. youth and young adults aged 13–24 years old showed a significant decline in sales of ECs in 2020 compared to 2019 (*p *< 0.001). Moreover, there was a significant decrease in mint flavor and an increase in tobacco-flavored ECs use (*p* = 0.001). Furthermore, a significant increase in disposable ECs use (*p* = 0.001) was observed among youths and young adults. Users of fruit-flavored ECs utilized disposable devices significantly more than other device types (*p* = 0.004) [47]. The gigantic increase in sales of sweet/fruit-flavored disposable ECs since 2020, especially among young people, raises concerns about the negative effects of their use by minors, including: brain development, nicotine dependence, willingness to quit smoking or combustible cigarettes smoking initiation [48, 49].

Smoking during adolescence is associated with impaired brain development (prefrontal cortex (PFC) synapse functioning) [50]. Nicotine acts directly on brain areas associated with emotional and cognitive processes (lower psychomotor speed and cognitive flexibility) [51, 52]. Moreover, smoking during adolescence leads to disturbances in attention and working memory and increases the risk of mental and behavioral problems (i.e., depressive disorders, agoraphobia, panic disorders, addictions, and antisocial personality disorders) [53, 54].

Analysis of blood samples showed significantly lower nicotine C_max_ of all studied ECs compared with TC (*p* < 0.05); however, no differences among ECs devices (*p* < 0.05) were determined [55]. In another study of adult smokers, there was no significant difference in plasma nicotine C_max 0–120_ level between BIDI® Stick ENDS with any flavor (concentration range: 15.3 ± 9.90–17.6 ± 9.00 ng/mL) and usual brand (UB) of cigarettes (mean concentration: 16.2 ± 9.17 ng/mL). Furthermore, there was no significant difference in AUC_0–120_ for BIDI® Stick ENDS with any flavor (concentration range 569.7 ± 327.29–628.6 ± 408.99 min*ng/mL) and UB cigarettes (mean concentration 747.1 ± 325.48 min*ng/mL) and in T_max 0–120_ (range 5–7 min for all flavors of BIDI® Stick ENDS and UB cigarettes (243.6 ± 179.04 min*ng/ml) [56]. Five min-post disposable cig-a-like ECs (CLs) exposure, the plasma nicotine level was 5.5 ng/mL (vs. 9.3 ng/mL for tank model (TM) ECs and 17.1 ng/mL for TC) [57]. Furthermore, disposable ECs may contain nicotine salts, present mostly in protonated form. This form of nicotine, compared to free base form, is correlated with a smooth sensory effect and higher nicotine absorption [58]. As we mentioned above, nicotine concentrations from using ECs can be just as high as with cigarette use, which may have negative consequences, especially for the adolescents who started using them as the first type of tobacco products [59].

Early exposure to nicotine is associated with high risk of nicotine dependence in adulthood [60]. Signs of nicotine dependence are frequently observed among daily high nicotine content pod-based ECs users (i.e., JUULs) [61]. However, 80.7% of disposable e-cigarette users aged 13–24 years old showed at least one sign of nicotine dependence [62]. There were no significant differences in the Penn State Electronic Nicotine Dependence Index (PSECDI) score (F(3,127) = 2.76, *p* = 0.045) and E-cigarette Dependence Scale (EDS-4) (F(3,127) = 1.64, *p* = 0.183) between EC device types [63]. In a cohort online survey of U.S. adults aged 19–20 years old, 24% of sweet/fruit-first-flavor users had at least one symptom of nicotine dependence compared with 22% of tobacco/flavorless/other-first-flavor users and 46% of menthol/mint-first-flavor users [64].

Studies demonstrated that early nicotine initiation reduces smoking cessation rate [65–67]. Two-arm, double-blind randomized clinical trial (RCT) of young adults, aged 21–35 current smokers (> 10 cigarettes per day (CPDs)) who had received 4.5% of nicotine disposable ECs or placebo for 3 weeks showed a significant reduction in CPDs at both study time periods (1 and 3 weeks) for disposable ECs (*p* < 0.001) and placebo (*p* < 0.001) groups (vs. baseline). Moreover, at week 3, significantly fewer CPDs were observed in the disposable ECs group than in the placebo group (*p* = 0.03) [68]. In RCT of cigarette smokers with serious mental illness (SMI) randomized to a control group or disposable e-cigarettes for 8 weeks, at weeks 2–8 there was a decrease in CPDs in the treatment (7.5 CPD; 95% CI = 5.9- 9.2) vs. control group (18.1 CPD; 95%CI = 16.4–19.8). Moreover, there was a decline in carbon oxide (CO) in the treatment group (16.4 ppm CO; 95%CI = 13.4- 19.5) compared with the control group (25.4 ppm CO; 95%CI = 22.4–28.9). At weeks 2–8, 19–22% of participants in the treatment group reported no smoking, compared with 0% of participants in the control group. At weeks 13–26, there was a significant reduction in CPDs in the treatment group compared with the control group, but not in the CO level [69]. Such results suggest that switching to disposable ECs may help to reduce CPDs. However, there is only limited evidence that ECs are effective as a smoking cessation tool. As data suggested, ECs may increase the tendency to use conventional and electronic cigarettes simultaneously [70, 71]. Furthermore, there is no data on smoking cessation rate among disposable ECs users exclusively.

Approximately 90% of adult smokers started using tobacco products before reaching 18 years old [72, 73]. A longitudinal population-based cohort study of students who started using ENDS showed a significantly lower odds ratio of starting using TC subsequent to ENDS initiation for disposable ECs as the initial device (vs. refillable ECs; OR = 0.42; 95% CI = 0.19–0.89, *p* = 0.03), but not significant in adjusted model (aOR = 0.47, 95% CI = 0.21–1.07, *p* = 0.07). Moreover, there was a significantly higher odds ratio for starting using TC in the same wave as starting ENDS for disposable ECs as the initial device (vs. refillable ECs; OR = 2.01; 95% CI = 1.01–4.03, *p* = 0.05), but not significant in adjusted model (aOR = 1.98; 95% CI = 0.97–4.04, *p* = 0.06) [74].

This study has several limitations. Firstly, small sample size (n = 4 studies) affects the reliability of the findings. In the included studies, there was also a small sample of never smokers/vapers (never-users of any tobacco products) who initiated vaping with sweet/fruit-flavored disposable ECs. Moreover, there was a limited number of data on the progression of using tobacco products (follow-up) by the participants. Furthermore, each analyzed study based on the self-reported tobacco product use behaviors, which increases the risk of response bias. Secondly, all of the analyzed data came from the USA, which makes it impossible to generalize obtained results to the wider global population. In addition, increased popularity of disposable ECs on US market was associated not only with aggressive marketing of such products but also by implementing the Food and Drug Administration reinforcement policy against certain unauthorized flavored EC products that appeal to youths, including fruit and mint flavors in 2020. The regulation had an impact on reducing sales of unauthorized flavored cartridge-based ECs, but it does not apply to disposable ECs. Moreover, not all publications analyzing the popularity of different flavors of liquids among recipients of different ages (adolescents, young adults and adults) referred to the division into different models of e-cigarettes (mod, box, pod, tank, disposable, etc.), which limited the number of publications included in this systematic review. Moreover, there is only a small number of publications which focus on never smokers of any tobacco products, and most of them investigated ever users. The heterogeneity of the results in terms of the large variety of disposable e-cigarettes available on the market means that some publications discuss the increase in interest in specific brands (one brand or several most frequently chosen ones), and not the entire group of disposable ECs.

## Conclusions

Due to limited number of studies, their limited location (US studies; no possibility of data generalization), small number of never smokers/vapers included in the studies, and moderate quality of the studies, it is difficult to clearly determine the effect of sweet/fruit-flavored disposable ECs on early nicotine initiation. Moreover, it is difficult to determine if sweet/fruit-flavored disposable EC have a role in avoiding the initiation of tobacco cigarettes or delaying this initiation by the effect of competition.

The findings from this systematic review are preliminary and require validation through high-quality, global studies among youth and young adult never-smokers/never-vapers who initiated using tobacco products with sweet/fruit-flavored disposable ECs and continue vaping and/or smoking any tobacco products (and any flavors).

## Supplementary Information

Supplementary Material 1.

## Data Availability

All data generated or analyzed during this study are included in this published article.

## Abbreviations

aOR: Adjusted odds ratio
aRR: Adjusted rate ratio
CAGR: Compound annual growth rate
CO: Carbon monoxide
CPD: Cigarette per day
EC: Electronic cigarette
EDS-4: E-cigarette Dependence Scale 4
ENDS: Electronic nicotine delivery system
FDA: Food and Drug Administration
HPHC: Hazardous and potentially hazardous constituent
PFC: Prefrontal cortex
PRISMA: Preferred Reporting Items for Systematic Reviews and Meta-Analyses
PSECDI: Penn State Electronic Nicotine Dependence Index
RCT: Randomized clinical trial
ROS: Reactive oxygen species
SMI: Serious mental illness
TC: Tobacco cigarette
TM: Tank model
UB: Usual brand
