# Supplementary material for: The role of sweet/fruit-flavored disposable electronic cigarettes on early nicotine initiation - a systematic review
Source: BMC Public Health. 2025 Feb 17;25:643. doi: 10.1186/s12889-025-21897-z (PMC11834490; doi:10.1186/s12889-025-21897-z)
Supplement: Supplementary file 1 — Supplementary Material 1. [file 12889_2025_21897_MOESM1_ESM.docx]

**Table 1 – Full search strategy for PubMed, Scopus, Web of Science and Science Direct databases**

| **Database** | **Search terms** | **Search results (July, 25 2024)** |
| --- | --- | --- |
| **PubMed** | (electronic AND nicotine AND delivery AND system OR ends OR disposable AND electronic AND cigarette OR disposable AND e-cigarette OR cig-a-like AND electronic AND cigarette OR cig-a-like AND e-cigarette) AND (nicotine AND initiation) | 934 |
| **Scopus** | (electronic AND nicotine AND delivery AND system OR ends OR disposable AND electronic AND cigarette OR disposable AND e-cigarette OR cig-a-like AND electronic AND cigarette OR cig-a-like AND e-cigarette) AND (nicotine AND initiation) | 523 |
| **Web of Science** | **Search 1:** (electronic AND nicotine AND delivery AND system OR ends OR disposable AND electronic AND cigarette OR disposable AND e-cigarette OR cig-a-like AND electronic AND cigarette OR cig-a-like AND e-cigarette) AND (nicotine AND initiation OR nicotine initiation) | 2.9 million + |
|  | **Search 2: (**ends OR disposable electronic cigarette OR disposable e-cigarette OR cig-a-like electronic cigarette OR cig-a-like e-cigarette) AND (nicotine initiation) | 422 |
| **Science Direct** | **Search 1:** (electronic nicotine delivery system OR ENDS OR disposable electronic cigarette OR disposable e-cigarette OR cig-a-like electronic cigarette OR cig-a-like e-cigarette AND nicotine initiation) | 534,177 |
|  | **Search 2:** (disposable electronic cigarette OR disposable e-cigarette OR cig-a-like electronic cigarette OR cig-a-like e-cigarette AND nicotine initiation) | 3341 |
